# Biomarker dynamics and prognosis in breast cancer after neoadjuvant chemotherapy

**DOI:** 10.1038/s41598-021-04032-x

**Published:** 2022-01-07

**Authors:** Cristina Zarotti, Bärbel Papassotiropoulos, Constanze Elfgen, Konstantin Dedes, Denise Vorburger, Bernhard Pestalozzi, Andreas Trojan, Zsuzsanna Varga

**Affiliations:** 1 Department of Pathology and Molecular Pathology, University Hospital Zurich, Schmelzbergstrasse 12, 8091 Zurich, Switzerland; 2 Breast Center Seefeld, Zurich, Switzerland; 3 Comprehensive Cancer Center, Breast Unit, University Hospital Zurich, Zurich, Switzerland; 4 Department of Gynecology, University Hospital Zurich, Zurich, Switzerland; 5 Department of Medicine, Cantonal Hospital Winterthur, Winterthur, Switzerland; 6 Department of Medical Oncology and Hematology, University Hospital Zurich, Zurich, Switzerland; 7 Onkozentrum Zurich, Zurich, Switzerland; 8 University of Witten-Herdecke, Witten, Germany

**Keywords:** Medical research, Epidemiology

## Abstract

Breast cancer is a biologically diverse disease with treatment modalities selected based on tumor stage and tumor biology. Distinct intrinsic subtypes and surrogate biomarker profiles play a major role for therapeutic decisions. Response rates to systemic and local treatments as well as the interaction with epidemiological risk factors have been validated in clinical trials and translational studies. This retrospective study addresses the question how biomarker profiles and treatment modalities in the neoadjuvant chemotherapy setting have changed during the past 15 years and what prognostic impact these changes implicate. 342 female breast cancer stage I-IV patients receiving neoadjuvant chemotherapy between 2003 and 2017 were analyzed. Overall survival (OS) was correlated with preoperative clinical stage, postoperative pathological stage, treatment modalities and tumor biology before and after chemotherapy. Two subgroups were separated using an arbitrary cut-off year at 2009/2010, due to 2010 when platinum containing regimens were first administered. Median follow-up was 54 months. 57 (17%) patients died; recurrences occurred in 103 of 342 (30%) patients. Nodal stage and intrinsic subtypes (pre- and postoperative) significantly correlated with OS (*p* < 0.001). Preoperative histological grading lacked prognostic power. When comparing the patient characteristics of the subgroups, we found significant difference in the following characteristics: cT, ypT, ypN, pCR and chemotherapy regimens (*p* < 0.001). There was no difference in OS when comparing the two subgroups. Pathological complete response (pCR) rates had a significant impact on OS and disease-free survival (DFS) in HER2+ and triple negative subtypes (*p* = 0.03). In multivariate analysis, high proliferation index (> 30%), clinical metastatic stage and pathological tumor stage had prognostic impact on OS (*p* < 0.001, *p* = 0.0001, *p* = 0.002). Clinico-pathological factors and distinct therapy regiments especially in triple negative and HER2+ subtypes have prognostic impact on pCR, OS and DFS after neoadjuvant chemotherapy.

## Introduction

Since the early years of the 1970s, neoadjuvant chemotherapy has been used to downstage locally advanced cancer to make it operable^1^. In more recent years, neoadjuvant chemotherapy has been increasingly used for operable (early) breast cancer because of the several advantages like improved rates of breast-conserving therapy, minimizing the need for axillary lymph node dissection and collecting information on chemosensitivity in-vivo including the possibility to switch therapy if the response is inadequate^2,3^. Obtaining a complete pathological response has been clearly shown to improve overall survival (OS)^2^.

In a large meta-analysis of the Early Breast Cancer Trialists Collaborative Group, several studies could confirm a similar mortality following adjuvant and neoadjuvant therapy^4^. Due to increased risk for metastasis of the HER2-receptor positive and triple negative subtypes, patients may also benefit from early treatment of possible distant micro-metastasis with neoadjuvant^5,6^.

Patients with pathologic complete response (pCR) after neoadjuvant chemotherapy have been shown to have improved disease-free survival (DFS) and overall survival (OS)^2,5,7^. pCR is defined as no invasive residual tumor (in some guidelines also including the complete absence of in situ carcinoma) in breast and lymph nodes after neoadjuvant^2^. Depending on the intrinsic breast-cancer subtypes, the incidence and prognostic impact of pCR can^2^ vary.

The distinct biological subtypes response differently to systemic and local treatments and differ in their epidemiological risk factors^8^. According to the 2011 St. Gallen consensus recommendations, tumors were classified into four different molecular subtypes: triple negative, HER2+, Luminal A und Luminal B like (HER2+ or enriched and HER2−^8^. Based on annual and bi-annual consensus definitions, the treatment guidelines for the different intrinsic subtypes have been adapted several times during the past decades^8^.

In the present study, we addressed the question, whether there is a prognostic difference in breast cancer patients treated with neoadjuvant chemotherapy prior to or after 2010 due to relevant changes in treatment strategies such as the use of platinum containing regimens. Additionally, we tested whether different intrinsic subtypes using the same cut-off year at 2010 have any impact on OS, DFS and pCR rates.

## Methods

### Patients’ characteristics

This retrospective study includes breast cancer patients treated between September 2003 and August 2017 at the University Hospital of Zurich, Switzerland or at the affiliated breast centers. Inclusion criteria for this study were that the patients were treated at the University Hospital of Zurich or at the affiliated centers along with available information on follow-up and therapies. More than 100 patients were excluded because information was missing on the chemotherapy or the patients were lost to follow-up.

Histopathological analyses of all samples were conducted in the Department of Pathology and Molecular Pathology of the University Hospital of Zurich. Clinical data regarding oncological treatment modalities were available in the four affiliated breast centers (Breast Center Seefeld Zurich, Breast Unit of the Comprehensive Cancer Center of the University Hospital Zurich, the Breast Center of the Regional Hospital Zollikerberg, Zurich and the Breast Center of the Regional Hospital Männedorf, Switzerland). Invasive breast cancer in all patients was confirmed in pre-therapeutic breast core or vacuum assisted biopsies. Clinical data included demographics, clinical tumor (cT) and nodal (cN) stage, assessment for metastases (M), pre-therapeutic histological grade (G), postoperative histopathological tumor size (ypT) and nodal state (ypN), hormone receptor and HER2 receptor status, Ki67 index, type of neoadjuvant chemotherapy and recurrence rate. According to the 2011 St. Gallen Consensus Conference, tumors were classified in four different molecular subtypes: triple negative, HER2+, Luminal A und Luminal B like (HER2+ or enriched and HER2−^8^. Intrinsic subgroups were defined as described in details in Table 1.

**Table 1:** Definitions of intrinsic subtypes of breast cancer.

Intrinsic subtype	Definition
Luminal A	Strongly ER and/or PR positive HER2 negative Ki-67 low
Luminal B (HER2 negative)	Variable degrees of ER and/or PR positive HER2 negative Ki-67 high
Luminal B (HER2 positive or enriched)	ER and/or PR positive HER2 positive Any Ki-67
HER2+	ER und PR absent HER2 positive
Triple negative	ER und PR absent HER2 negative

ER, oestrogen receptors; PR, progesterone receptors; HER2, human epidermal growth factor receptor 2.

### Assessment of intrinsic factors (hormone receptor and HER2 status, Ki67 index)

All laboratory procedures and scoring criteria for the assessment of the four intrinsic factors have been carried out in an identical way as they previously described according to timely current guidelines and laboratory procedures^9–11^.

### Therapy regiments

The neoadjuvant chemotherapy regimens were very heterogenous in the individual patients due to the real-life data. Most of the patients (90%) received a chemotherapy containing taxane, 75% of the patients were treated with a combination with taxane and anthracycline. 24% had a chemotherapy containing platinum and 31% received an anti HER2-therapy. The neodjuvant chemotherapy was assigned in subgroups as follows: containing an anti-HER2-antibody; containing platinum; containing platinum and anti-HER2-antibody; others (with no platinum or anti-HER2-antibody). Endocrine therapy was not assessed separately in this study.

### Statistical methods

The primary endpoint was overall survival (OS). The secondary endpoint of this study was disease free survival (DFS), which was defined as the time of breast cancer recurrence (local, regional or distant). The period between diagnosis to the first event defined the duration of DFS. The date of recurrence was determined as the first clinically or pathologically confirmed incidence of relapsing invasive breast cancer. OS and DFS was analyzed by using the Kaplan–Meier test, log-rank and Cox regression methodologies. Hazard ratios (HR) with 95% confidence interval (CI) were calculated using the Cox regression test. *p* values < 0.05 were considered as statistically significant. The statistical software IBM SPSS Statistics (Version 26.0, Armonk, NY: IBM Corp., USA) was purchased and used to perform all statistical analyses.

### Ethical approval

This study is a part of a larger breast cancer project, previously approved by the Ethical commission of the Canton Zurich (KEK-ZH-2012-553). Data collection was carried out via electronic medical records and entered in an anonymized databank. All analyses were performed in accordance with the relevant guidelines and regulations.

### Consent for publication

Informed consent to use clinical data for research has been provided by the patients.

## Results

### Clinical characteristics

A total of 342 female breast cancer patients treated with neoadjuvant chemotherapy were reviewed in the observational period. The median follow-up time was 46 months (mean 54, range 5–168 months). Recurrence of disease occurred in 103 of 342 patients (30%), 57 of 342 (17%) patients died during the observation period. The average overall survival was 134 months (95% CI 125–142), the disease-free survival 108 months (95% CI 99–117). Median age was 49 years (range 25–81).

Clinical and histopathological data in univariate analysis are presented in Table 2.

**Table 2:** Univariate analysis: overall survival in correlation with clinico-pathological parameters, applied chemotherapy regimens, intrinsic subtypes und subgroups in pre-therapeutic biopsy specimens.

	N (percentage) = 342 (100%)	HR (95%CI) of overall survival	*p* value
**Age (median, range)**	49 (25–81)	1.02 (CI 1.00–1.05)	**0.015**
**cT stage**			
cT1	55 (16%)	1.0	**0.003**
cT2	168 (49%)	3.52 (CI 0.83–14.98)	
cT3-4	118 (35%)	6.62 (CI 1.56–27.73)	
**ypT stage**			
ypT0	107 (31.3%)	1.0	**<** **0.001**
ypT1	107 (31.3%)	1.92 (CI 0.73–5.07)	
ypT2	75 (22%)	3.32 (CI 1.29–8.49)	
ypT3-4	52 (15.1%)	6.56 (CI 2.62–16.45)	
NA	1 (0.3%)		
**cN stage**			
cN0	88 (25.7%)	3.49 (CI 1.39–8.75)	**0.005**
cN1-3	248 (72.5%)		
NA	6 (1.8%)		
**ypN stage**			
ypN0	176 (51.5%)	4.65 (CI 2.38–9.12)	**<** **0.001**
ypN1-3	131 (38.3%)		
NA	35 (10.2%)		
**cM stage**			
cM0	293 (85.6%)	5.88 (CI 3.39–10.19)	**<** **0.001**
cM1	46 (13.5%%)		
NA	3 (0.9%)		
**Preoperative histological grading**			
G1/2	131 (38.3%)	1.38 (CI 0.78–2.44)	0.27
G3	188 (55.0%)		
NA	23 (6.7%)		
**Proliferation Index Ki67**			
Low (< 30%)	110 (32.1%)	3.01 (CI 1.46–6.21)	**0.002**
High (≥ 30%)	68 (19.9%)		
NA	164 (48.0%)		
**Pathologic complete response**			
No	239 (70%)	0.15 (CI 0.05–0.47)	**<** **0.001**
Yes	103 (30%)		
**Subtype**			
Luminal A	75 (21.9%)	1.0	**<** **0.001**
Luminal B Her2+	72 (21.1%)	0.66 (CI 0.23–1.99)	
Luminal B Her2−	44 (12.9%)	3.79 (CI 1.68–8.51)	
HER2+	60 (17.5%)	1.02 (CI 0.36–2.86)	
Triple Negative	90 (26.3%)	2.65 (CI 1.19–5.89)	
NA	1 (0.3%)		
**Chemotherapy**			
HER2-antibody therapy	94 (27.5%)	1.0	**0.043**
Platinum	64 (18.7%)	2.75 (CI 1.06–7.10)	
Others	164 (48.0%)	2.64 (CI 1.17–5.95)	
Combination of antibody and platinum	19 (5.5%)	0.67 (CI 0.08–5.50)	
NA	1 (0.3%)		
**Subgroups**			
2003–2009	80 (23%)	0.74 (CI 0.42–1.33)	0.312
2010–2017	262 (77%)		

*p* values < 0.05 (bold labeled) are significant.

NA, not available.

### Correlation between OS and nodal / tumor and metastatic stage

**Figure 1:**
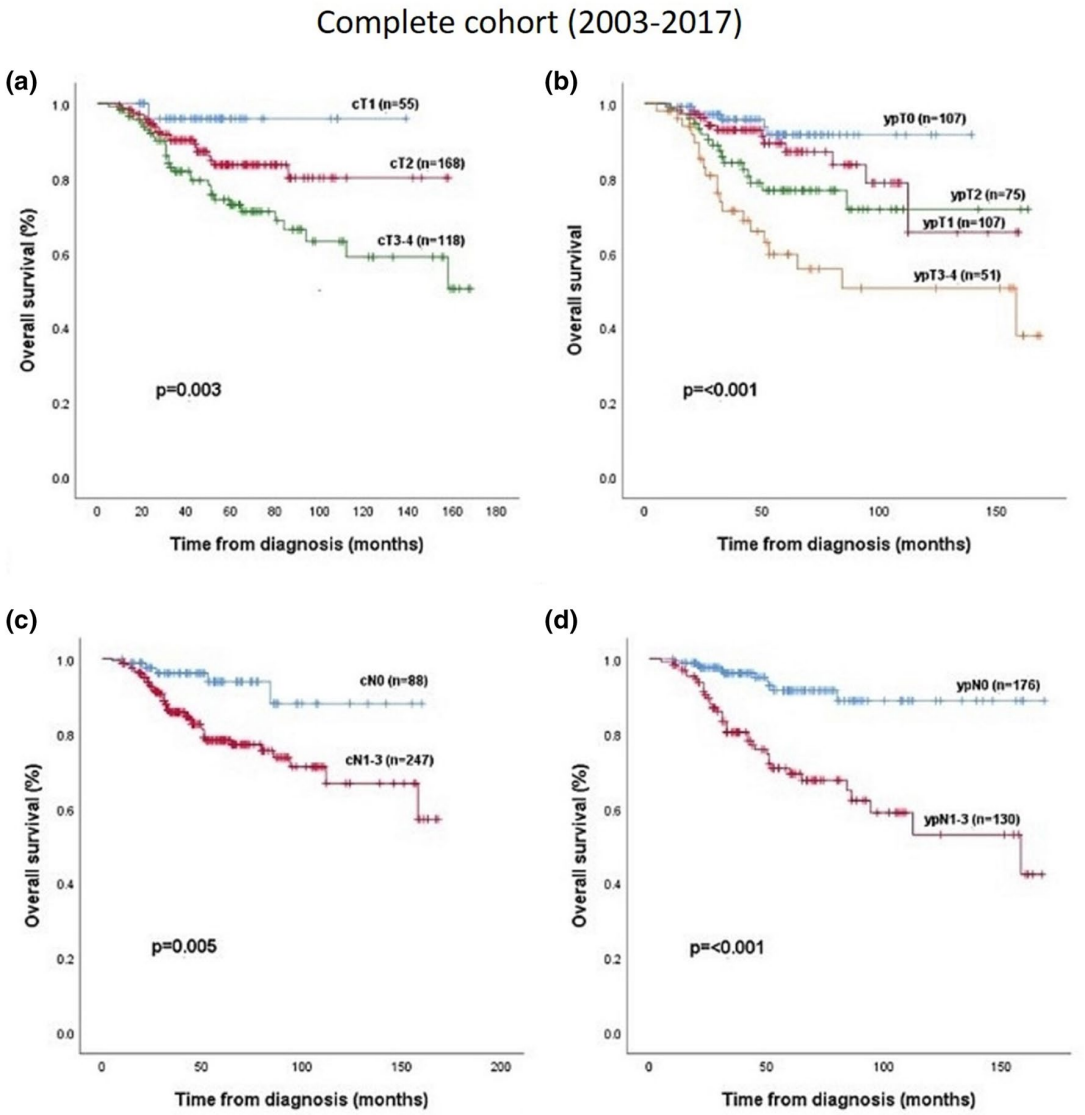
Overall survival in relation to (**a**) cT-stadium, (**b**) ypT-stadium, (**c**) cN-stadium and (**d**) ypN-stadium in the whole cohort (2003–2017).

Most tumors were classified as cT2 (49%), whilst cT1 were 16% and cT3-4 were 35%. The clinical tumor size prior to therapy significantly affected prognosis and overall survival when we grouped the tumor into the categories cT1, cT2 versus the larger cT3-4 tumors (*p* = 0.003) (Fig. 1a).

After completing the neoadjuvant chemotherapy, no residual tumor (ypT0) or small residual tumor (ypT1) was present in one third of the patients (each 31.3%). 22% had ypT2 and 15.1% ypT3-4 post-therapeutic stage. The residual tumor size had a significant prognostic impact on OS (*p* < 0.001) (Fig. 1b).

Only 88 of 342 (25.7%) patients were diagnosed with preoperative negative nodal status, 248 of 342 (72.5%) were node positive (cN1-3 status) at date of diagnosis. The preoperative nodal stage (cN0 vs cN1) had a significant effect on OS (*p* = 0.005) (Fig. 1c). After completing the neoadjuvant chemotherapy almost two thirds (58%) of the patients had a node negative status. Any nodal positive ypN+ status had a significantly worse prognosis compared with a node negative (ypN0) status (*p* < 0.001) (Fig. 1d).

**Figure 2:**
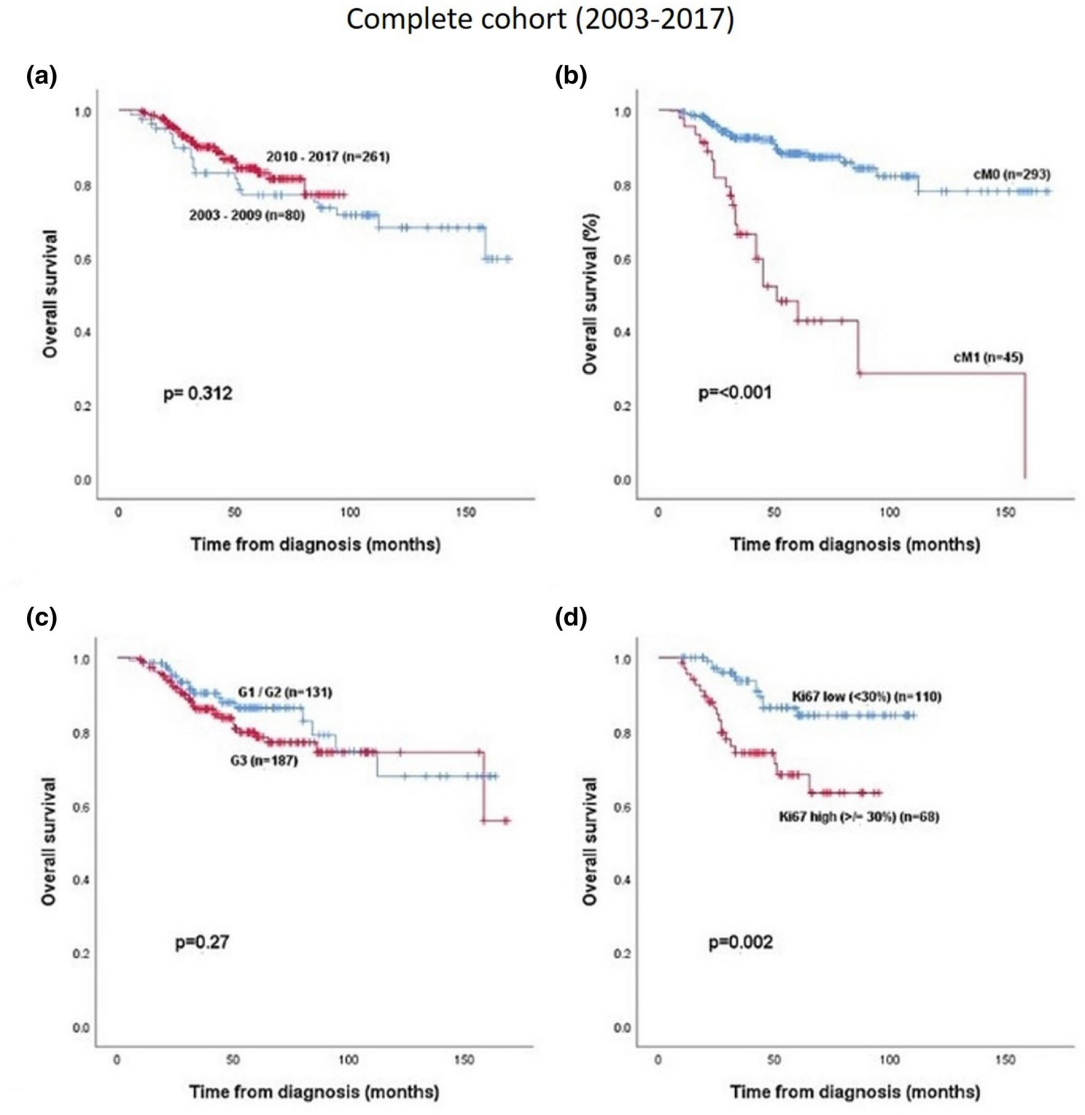
Overall survival in relation to (**a**) subgroup (2003–2009 vs 2010–2017), (**b**) cM-stage, (**c**) preoperative histological grading and (**d**) preoperative Ki67 proliferation index.

There was no prognostic difference in OS when patients were divided in two chronological subgroups. Subgroup 1 consisted of patients with diagnosis of breast cancer between 2003–2009 and subgroup 2 between 2010 and 2017 (Fig. 2a).

At the time of the preoperative diagnosis 46 (13.5%) of the patients had metastatic disease. OS in the non-metastatic group was significantly better than in the metastatic group (*p* < 0.001) (Fig. 2b).

### Histological characteristics, pre-therapeutic grading / Ki67 and OS

Most breast cancers were of invasive ductal type, NST (81%), 5% were of lobular type and 14% were mixed or special type.

Only 3% of all tumors were preoperative histological grade G1, 38.3% were of moderate grade G2 und two third (55%) were poor grade G3. Histological grading had no significant impact on OS, when G1 and G2 were compared with G3 tumors (*p* = 0.27) (Fig. 2c).

The preoperative proliferation-index Ki67 was available in 178 of the 342 patients. 110 (32.1%) had a low Ki67 index (defined as < 30%). The group with a high proliferation index (≥ 30%) had a significant worse prognosis than the group with low Ki67 index (*p* = 0.002) (Fig. 2d).

### Subgroup analysis (2003–2009 vs 2010–2017)

#### Correlation between OS and nodal/tumor and metastatic stage

For subgroup analysis we used 2009/20210 as a cut-off year, because platinum containing regimens were used since in 2010. Subgroup analysis showed prognostic differences between the two groups.

**Figure 3:**
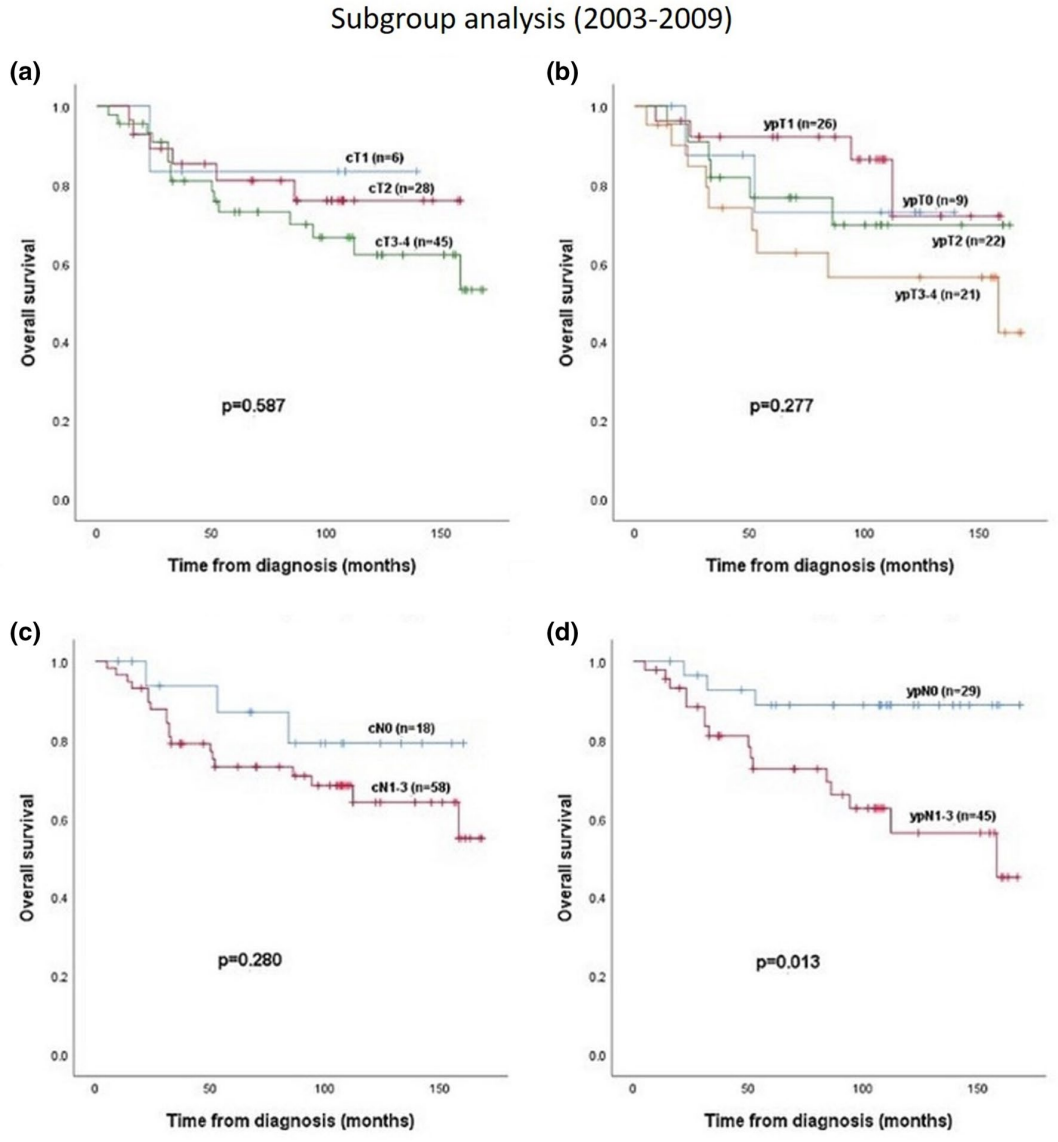
Overall survival in subgroup 1 (2003–2009) in relation to (**a**) cT-stadium, (**b**) ypT-stadium, (**c**) cN-stadium and (**d**) ypN-stadium.

2003–2009 (n = 80): Only postoperative nodal status correlated with OS (*p* = 0.013), preoperative nodal and tumor stage and postoperative nodal status did not have prognostic impact (Fig. 3).

**Figure 4:**
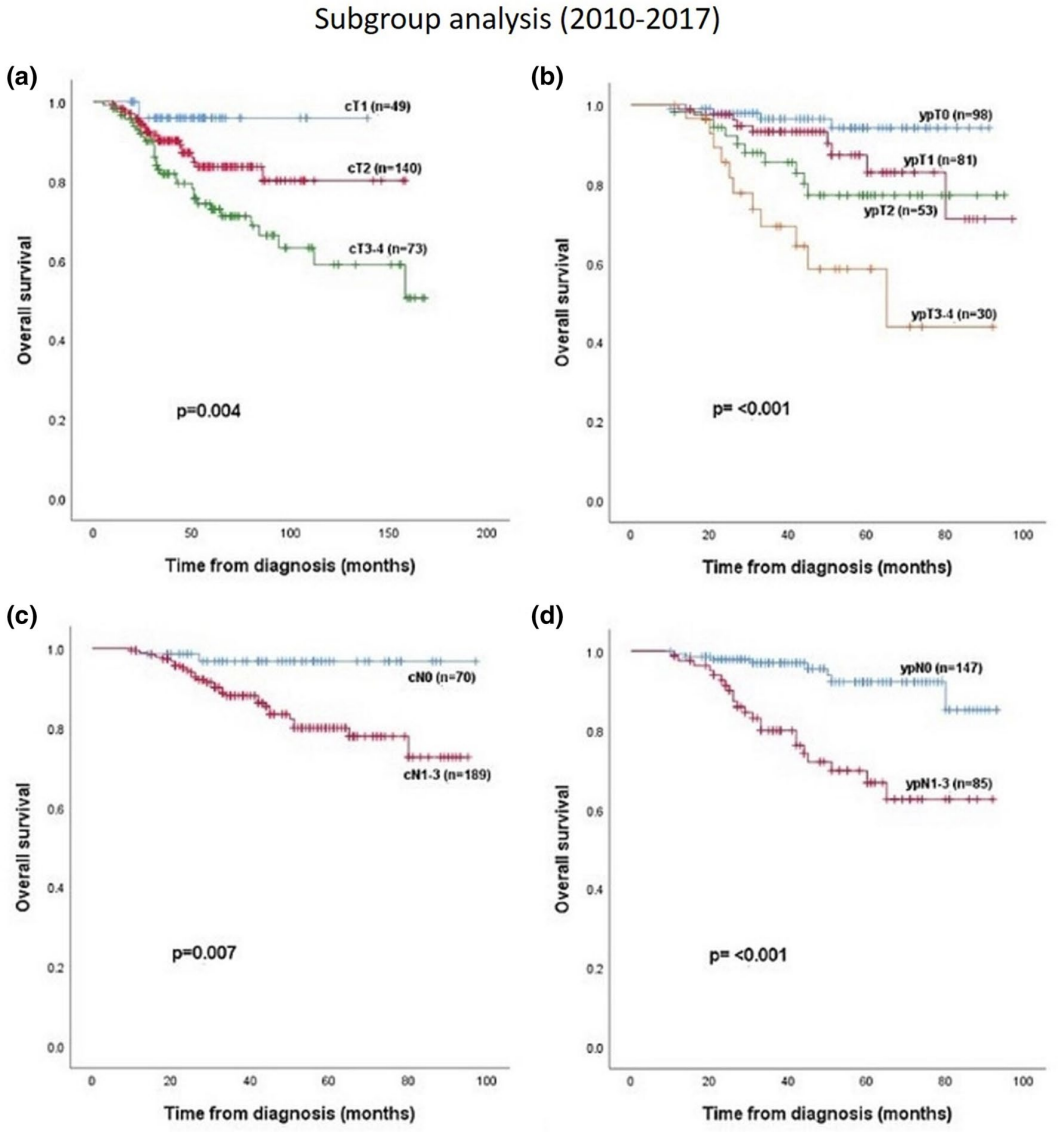
Overall survival in subgroup 2 (2010–2017) in relation to (**a**) cT-stadium, (**b**) ypT-stadium, (**c**) cN-stadium and (**d**) ypN-stadium.

2010–2017 (n = 262): Both pre- and postoperative nodal and tumor status had significant impact on OS (*p* = 0.004, *p* < 0.001, *p* = 0.007, *p* < 0.001) (Fig. 4).

#### Clinical and pathological staging , therapy regiments and PCR

When comparing clinical and pathological characteristics of the two subgroups, a significant difference was observed in the following characteristics: cT, ypT, ypN, pCR and chemotherapy regiments (Table 3).

**Table 3:** Patients’ characteristics in the two subgroups (2003–2009 vs 2010–2017).

Characteristics	2003–2009 (N = 80, 23%)	2010–2017 (N = 262, 77%)	*p* value
**Age (median, range)**	49 (26–79)	49 (25–81)	1.00
**cT stage**			
cT1	6 (7.5%)	49 (18.7%)	**<** **0.001**
cT2	28 (35%)	140 (53.4%)	
cT3-4	46 (57.5%)	73 (27.9%)	
**ypT stage**			
ypT0	9 (11.4%)	98 (37.4%)	**<** **0.001**
ypT1	26 (32.9%)	81 (30.9%)	
ypT2	22 (27.8%)	53 (20.2%)	
ypT3-4	22 (27.8%)	30 (11.5%)	
NA (not available)	1		
**cN stage**			
cN0	18 (23.4%)	70 (27%)	0.475
cN1-3	59 (76.6%)	189 (73%)	
NA	3	3	
**ypN stage**			
ypN0	29 (38.7%)	147 (63.4%)	**<** **0.001**
ypN1-3	46 (61.3%)	85 (36.6%)	
NA	5	30	
**cM stage**			
cM0	63 (80.8%)	230 (88.1%)	0.108
cM1	15 (19.2%)	31 (11.9%)	
NA	2	1	
**Histological grading**			
G1/2	32 (47.1%)	99 (39.4%)	0.311
G3	36 (52.9%)	152 (60.6%)	
NA	12	11	
**Proliferation Index Ki67**			
Low (< 30%)	19 (86.4%)	91 (58.3%)	**0.028**
High (≥ 30%)	3 (13.6%)	65 (41.7%)	
NA	58	106	
**Pathologic complete response**			
No	72 (90%)	167 (63.7%)	**<** **0.001**
Yes	8 (10%)	95 (36.3%)	
**Subtype**			
Luminal A	23 (29.1%)	52 (19.9%)	0.069
Luminal B Her2+	14 (17.7%)	58 (22.2%)	
Luminal B Her2−	15 (19.0%)	29 (11.1%)	
HER2+	13 (16.5%)	47 (18.0%)	
Triple negative	14 (17.7%)	76 (28.8%)	
NA	1		
**Chemotherapy**			
HER2-R antibody therapy	16 (20%)	78 (29.9%)	**<** **0.001**
Platinum	1 (1.2%)	62 (23.7%)	
Others	63 (78.8%)	102 (39.1%)	
Combination of antibody and platinum	0 (0%)	19 (7.3%)	
NA		1	

*p* values < 0.05 (bold labeled) are significant.

### Prognostic effect of therapy modalities on OS

**Figure 5:**
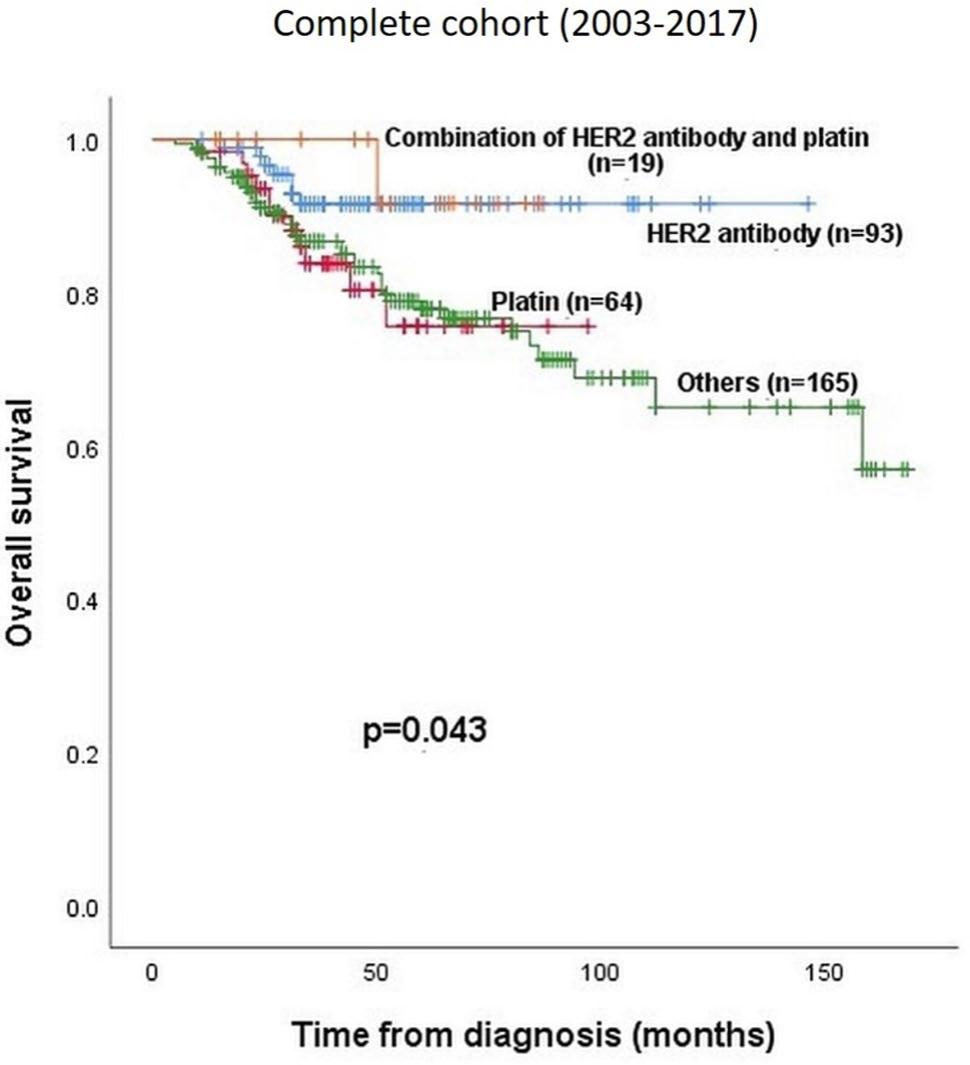
Overall survival in relation to the different chemotherapy strategies.

Preoperative chemotherapy with a monoclonal HER2 antibody was administered in 27% of the cohort, 19% had a platinum containing chemotherapy, 6% had a combination therapy with platinum and a monoclonal HER2 antibody and 48% received different therapy (neither platinum nor a HER2 antibody containing therapy). There was a significantly better OS for the HER2 antibody containing chemotherapy group compared to the other chemotherapy groups (with or without platinum) (*p* = 0.043). Table 2, Fig. 5.

### Intrinsic subtypes, OS and pathological response rate

103 (30%) of the patients had a pathological complete response. These patients had a significantly better prognosis (*p* = 0.001) (Table 2). The different intrinsic subtypes were associated with significantly different OS in the subgroups with pCR: triple negative (*p* ≤ 0.001) and HER2+ (*p* = 0.03) patients had better OS than luminal subtypes (Table 4). The pCR rates in the different subtypes was higher in the second subgroup 2010–2017 as in the earlier subgroup 2003–2009 in absolute numbers (Table 5). However, because of the small number of cases in each subtype, no Kaplan–Meier analyses were possible.

**Table 4:** Pathological complete response (pCR), overall survival (OS) and disease free survival rates (DFS) rates among the different subtypes overall and in subgroups.

Intrinsic subtype on pre-chemotherapy biopsies	Pathologic complete response (pCR)	pCR rates in %	Overall survival (%)	Disease free survival (%)	*p* value
Luminal A	pCR (n = 4)	5.3	100	100	0.426 (OS)
	no pCR (n = 71)		87.3	71.8	0.243 (DFS)
Luminal B HER2+	pCR (n = 25)	35.2	100	100	0.22 (OS)
	no pCR (n = 46)		89.1	71.7	**0.037 (DFS)**
	NA (n = 1)				
Luminal B HER2−	pCR (n = 6)	13.6	83.3	66.6	0.51 (OS)
	no pCR (n = 38)		57.9	44.7	0.85 (DFS)
HER2+	pCR (n = 26)	43.3	100	88.5	**0.03 (OS)**
	no pCR (n = 34)		82.3	44.1	**0.03 (DFS)**
Triple negative	pCR (n = 42)	47.2	95.2	92.3	**<** **0.001 (OS)**
	no pCR (n = 47)		63.8	51.1	**<** **0.001 (DFS)**
	NA (n = 1)				

*p* values < 0.05 (bold labeled) are significant.

**Table 5:** Association between pathological complete response (pCR) and breast cancer intrinsic subtype.

		Luminal A (incidence/total cases)	Luminal B HER2+ (incidence/total cases)	Luminal B HER2− (incidence/total cases)	HER2+ (incidence/total cases)	Triple negative (incidence/total cases)
2003–2009	pCR	4.3% (1/23)	7.7% (1/13)	6.7% (1/15)	30.8% (4/13)	7.1% (1/14)
2010–2017	pCR	5.8% (3/52)	41.4% (24/58)	17.2% (5/29)	46.8% (22/47)	54.7% (41/75)
2003–2017	pCR	5.3% (4/75)	35.2% (25/71)	13.6% (6/44)	43.3% (26/60)	47.2% (42/89)

### Intrinsic subtypes in pre- and post-chemotherapy specimens

**Figure 6:**
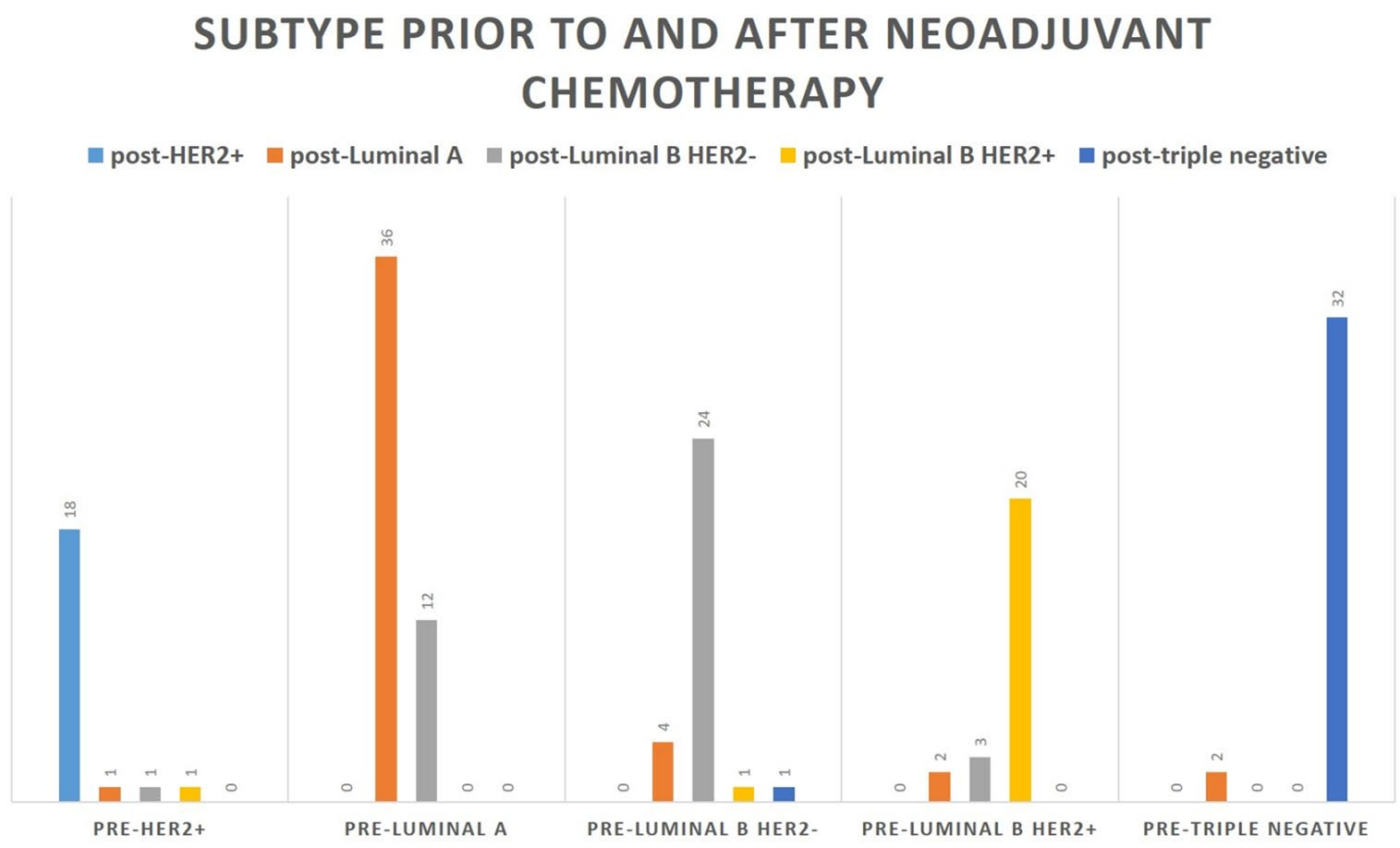
Biomarker changes after neoadjuvant chemotherapy in each intrinsic subtype.

The histopathological examination of the pre-chemotherapy breast biopsies showed, that 26.3% of the patients had a triple negative subtype, 21.9% luminal A subtype, 21.1% luminal B HER2+ subtype, 17.5% HER2+ subtype and 12.9% luminal B HER2− subtype (Table 2). The comparison of intrinsic subtypes between pre- and postoperative samples showed some slight variations in the frequency (between 6 and 25%), although the differences were not statistically significant (Fig. 6, Table 6).

**Table 6:** Changes in intrinsic subtype after neoadjuvant chemotherapy. Differences between pre- and post-chemotherapy assessments were not statistically significant.

	Subtype post-chemotherapy					Total
	HER2+	Luminal A	Luminal B HER2−	Luminal B HER2+	Triple negative	
Subtype pre-chemotherapy						
HER2+	18	1	1	1	0	21
Luminal A	0	36	12	0	0	48
Luminal B HER2−	0	4	24	1	1	30
Luminal B HER2+	0	2	3	20	0	25
Triple Negative	0	2	0	0	32	34
Total	18	45	40	22	33	158

### Multivariate analysis between clinic-pathological characteristics and OS

A multivariate analysis was conducted using backward stepwise variable selection to see if there are independent prognostic factors with impact on OS. The results are shown in Table 7. Accordingly, significant negative prognostic factors are Ki67 proliferation > 30% index (*p* < 0.001) as well as clinical metastatic stage (*p* = 0.001) and pathological ypT stage (*p* = 0.002).

## Discussion

In this retrospective study we could show, that real-life data of neoadjuvant chemotherapy treatment of breast cancer reliably reflect results of randomized clinical trials and adapted recommendations of clinical guidelines. Our data show, that clinico-pathological factors and distinct therapy regiments especially in triple negative and HER2+ subtypes have prognostic impact on pCR, OS and DFS after neoadjuvant chemotherapy also outside the clinical trial setting.

### Neoadjuvant chemotherapy

Neoadjuvant preoperative chemotherapy became the standard care in locally advanced breast cancer for downstaging the tumor in order to make it operable^3^. The role and clinical implications of neoadjuvant chemotherapy in early (or operable) breast cancer have been the subject of several studies since the early 1980s^3^. Our study data show that the number of patients treated with neoadjuvant chemotherapy has steadily increased over the past few years. In the subgroup between 2003 and 2009 only 80 patients were treated with neoadjuvant chemotherapy, from 2010 to 2017 a total of 261 patients received this therapy option obviously as a result of adjusted new treatment recommendations among others by the St. Gallen International Expert Consensus^8,12^.

### Intrinsic Subtypes

The incidence of intrinsic subtypes in our study is slightly different compared with other studies (Table 8). Especially, the luminal A and luminal B HER2− subtypes had different frequencies in our study when compared with previously published literature data. It is likely that these differences are due to high inter-and intraobserver variability in Ki67 labelling index. The incidences of the other subtypes were quite similar to existing literature data^2,7,13,14^. Especially, the higher incidence rates of the triple negative breast cancer when comparing our data from 2003 to 2009 with those from 2010 to 2017 corroborate with literature data^15,16^. The phenomenon of variable changes in biomarker profile is well documented in the literature, most likely due to clonal selection but also to sample variation and to laboratory variables^17–24^.

**Table 7:** Multivariate analysis of clinico-pathological data and overall survival (OS) by backward stepwise variable selection (significant results only).

	*p* value	HR (95% CI)
**Proliferation index Ki67**		
Low (< 30%)	< 0.001	5.44 (CI 2.16–13.71)
High (≥ 30%)		
**cM stage**		
cM0	0.001	4.97 (CI 1.97–12.75)
cM1		
**ypT stage**		
ypT0	0.002	1.0
ypT1		0.05 (CI 0.01–0.34)
ypT2		0.04 (CI 0.01–0.24)
ypT3-4		0.23 (CI 0.04–1.18)

**Table 8:** Incidence rates of the different intrinsic subtypes in comparison with other studies.

	Total number of patients	Luminal A (%)	Luminal B (%) HER2+	Luminal B (%) Her2−	HER2+ (%)	Triple negative (%)
2003–2009	78	29.5	16.7	19.2	16.7	17.9
2010–2017	262	19.9	22.1	11.1	17.9	29.0
2003–2017	340	22.1	20.9	12.9	17.6	26.5
German 2013^2^	1604	35.6	17.5	13.1	11.1	22.5
German 2020^13^	4193	39.0	17.9	8.5	12.8	21.7
CTNeoBC 2014^25^	5694	34.8	19.1	11.1	14.7	20.3
EORTC 2013^14^	1289	40	18.4	11.9	9.9	19.8

### pCR

Several previous studies provide evidence that patients who achieve a pathological complete response defined as ypT0 ypN0 have an improved survival^2,7,13^. Especially in aggressive breast cancer subtypes, such as triple negative and HER2 enriched carcinomas, pCR is a suitable surrogate end point, however, pCR is not a reliable marker for endpoint in luminal B HER2− or luminal A tumors^13^. This was confirmed in the CTNeo pooled analysis where pCR could not be validated as a surrogate endpoint for improved DFS and OS in these subgroups^7^.

In our study pCR rates have a significant predictive value regarding the DFS and OS for the following subtypes: HER2+ (non-luminal) and triple negative carcinomas. For the other subtypes pCR was not a prognostic factor for OS or DFS. Luminal A and luminal B HER+ tumours have been shown to have a better outcome than the other subtypes, luminal B HER- tumours had worse outcomes independently of pCR (Table 4).

Similar observations have been described previously in a large meta-analysis from Houssami et al. providing evidence for the strong association between the different intrinsic subtypes and the odds of achieving pCR^26^. The highest odds (5 to 7 times higher) for achieving pCR were demonstrated in two subtypes: HER2 enriched (non-luminal) and triple negative breast cancers, and approximately 19% of all patients treated with neoadjuvant chemotherapy achieved pCR^26^. In our study, 30% of our patients achieved pCR which is higher than previously reported and is most likely due to the higher representation of HER2 positive and triple negative subtypes in our cohort.

Furthermore, our subgroup analysis using the arbitrary cutoff at 2009/2010 indicates that pCR rates were considerably higher in the triple negative and the HER2-enriched subtypes in the years 2010–2017 when compared with the earlier period of 2003–2009. On one side, this observation might be related to the platinum-containing chemotherapy regimens for triple negative breast cancer, which was administered only in one patient in the subgroup from 2003 to 2009 and was often applied in the subgroup from 2010 to 2017^27,28^. On the other hand, the more frequent use of the HER2-targed therapy (trastuzumab and pertuzumab) in the subgroup from 2010 to 2017 would at least partially explain the higher pCR rate in the HER2+ patients^29,30^. The rather heterogeneous and not standardized administration of further chemotherapy regiments such as taxane or anthracyclins in our cohort do not facilitate to draw any additional causal conclusion on pCR in other intrinsic subgroups.

### Overall survival

As expected, we found significantly improved OS and DFS rates in patients with low tumor stage (cT or ypT), negative nodal status (cN0 and ypN) and in the absence of metastatic disease. In the early subgroup of 2003–2009, only the ypN status correlated with improved OS (*p* = 0.013). On the contrary, in the period of 2010–2017, several further clinic-pathological factors such as cT, ypT, cN and ypN stages had prognostic impact on OS. One reason for this result is most likely the relatively low patient number in this subgroup compared with the later cohort from 2010 to 2017. Moreover, the increasing use of neoadjuvant chemotherapy after 2010 and less use of adjuvant chemotherapy in high risk situations probably also explain these findings.

When comparing the patient characteristics of the two study groups, we found significant difference in several clinico-pathological characteristics such as cT, ypT, ypN, pCR and chemotherapy regimens, although these factors did not have any impact on OS in the subgroups. A reason for improved pCR rates without consecutive improved OS could lay in the uneven patients’ distribution respectively in the low patient’s number in the early subgroup from 2003 to 2009. Also, the shorter follow-up time in the second groups and the unbalanced distribution of intrinsic subtypes in both subgroups may have impact the data on OS. There were more triple negative cancers with unfavorable outcome and less Luminal A cancers with better outcome in the 2010–2017 subgroup than in the early group.

Our study further supports previous literature data, that overall survival in the triple negative subgroup from 2010 to 2017 is more favorable compared with the subgroup from 2003 to 2009^27–31^. This is most likely related to the platinum containing chemotherapy, which has been used since 2010 regularly for triple negative breast cancers^27–31^. The standard use of platinum-based chemotherapy for triple negative cancers have been a debate of controversial discussions^27,28,31–33^. According to the 2019 St. Gallen Consensus, the standard use of platinum-based chemotherapy in triple negative breast cancer is only recommended for patients with a high risk clinical situation (high tumor load, poor response to first two cycles of chemotherapy)^32^. Other opinions are in favor of platinum-containing therapy regime in all patients because of a high benefit in achieving pCR rate^32^. Several further studies and meta-analyses confirm a significantly increased pCR rate in triple negative tumors after platinum-based chemotherapy, even though worse hematological toxicities have been also reported^27,28,31,32^. Similarly to these data, in our study, we also observed higher pCR rate with improved OS and DFS in in the triple negative tumours in the subgroup from 2010 to 2017^27,28,31^.

Regarding HER2 positive disease, it has been described previously that pCR rates are surrogate endpoints for patients with HER2 positive tumors and patients receiving a HER2 antibody therapy achieve higher pCR rates and improved overall survival, which is not seen in patients without pCR and who are at high risk for relapse^29,30^. In our study, we observed a similar tendency difference in the two subgroups. In the group from 2010 to 2017 the use of trastuzumab or pertuzumab (or the combination of both antibodies) was much more common than in the subgroup from 2003 to 2009 and HER2 positive patients also had considerably higher pCR rates and better OS.

## Conclusions

Our retrospective cohort study confirms improved OS and pCR in HER2 positive and triple negative subtypes in a real-life patients’ cohort. These findings corroborate with previous findings that the use of combined anti HER2 treatment as well as the addition of a platinum drug to neoadjuvant chemotherapy is beneficial for breast cancer patients with these subtypes. Differently to most of the previous studies, which were randomized trials addressing predictive and prognostic values of different therapy strategies, our study is based on real-life data outside the clinical trial setting show how the outcome changed over the past years after therapy strategies were adjusted. Our study shows exactly how these treatment strategies work in “real-life” and not only in randomized trials. Therefore, it is important to validate the results of randomized clinical trials using real-life data outside the clinical trial setting and to confirm whether data from clinical trials can be applied for a wide patient population outside the clinical trial setting.

Additionally, biomarker dynamics and prognosis in regular clinical setting over two decades exactly reflect the improvement on new therapy regiments in the neoadjuvant setting.

However, because of the “real-life” data the chemotherapy regimens were very heterogenous and the study is a retrospective analysis and these results have a strong prognostic value but lack direct predictive evidence. Further research with prospective randomized controlled studies is needed to confirm these results.

## Data Availability

All data are available upon request to the corresponding author without restrictions.
